# The Effect of Sickle Cell Disease on Seizure-Related Hospitalizations: An Analysis of the Nationwide Inpatient Sample 2021

**DOI:** 10.7759/cureus.86025

**Published:** 2025-06-14

**Authors:** Anushareddy Muddasani, Anudeep Surendranath, Ankur Varma

**Affiliations:** 1 Internal Medicine, University of Arkansas for Medical Sciences, Little Rock, USA; 2 Neurology, CHI St. Vincent Hot Springs, Hot Springs, USA; 3 Hematology and Medical Oncology, University of Arkansas for Medical Sciences, Little Rock, USA

**Keywords:** in-hospital mortality, national inpatient sample database, seizures, sickle cell disease (scd), stroke

## Abstract

Objective: This study aims to evaluate the effect of sickle cell disease (SCD) as a comorbidity on seizure-related hospitalizations, with a focus on demographic disparities, clinical characteristics, and outcomes.

Methods: This retrospective cohort study utilized the 2021 National Inpatient Sample (NIS) to identify patients admitted with a principal diagnosis of seizures. Patients were stratified into two groups based on the presence or absence of SCD as a comorbidity. Primary outcomes included in-hospital mortality, while secondary outcomes included hospital length of stay and total hospital charges. Multivariate logistic and linear regression models were used to adjust for confounders.

Results: Among 263,625 patients hospitalized for seizures, 434 (0.17%) had a comorbid diagnosis of SCD. Patients with SCD were younger (mean age: 39.02 vs. 44.1 years, p < 0.05) and predominantly African American (78.82% vs. 22.76%, p < 0.05). They also had a higher Charlson Comorbidity Index (CCI) score and were more likely to have an ischemic stroke (4.44% vs. 1.16%, p < 0.05). However, after adjusting for confounders, SCD was not significantly associated with an increased in-hospital mortality (adjusted OR: 1.60, 95% CI: 0.22-11.44, p = 0.637), length of stay (p = 0.825), or total hospital charges (p = 0.827).

Conclusion: Despite notable demographic and clinical differences, the presence of SCD as a comorbidity did not significantly impact in-hospital mortality, length of stay, or hospital charges in seizure-related hospitalizations.

## Introduction

Sickle cell disease (SCD) is an inherited hemoglobinopathy characterized by chronic hemolysis, vaso-occlusive crises, and multi-organ complications. Neurological complications, including both clinical and silent cerebral infarcts, are well-documented in SCD and significantly contribute to morbidity and mortality [1]. While epilepsy and seizures are reported to be more common in individuals with SCD than in the general population, they remain understudied compared to other complications [2]. In a nested case-control study using data from the Cooperative Study of Sickle Cell Disease, the prevalence of seizures was found to be 5.5% in pediatric patients and 9.0% in adult patients, underscoring the significant neurological burden associated with the condition [3]. Seizures in SCD are not only a marker of increased morbidity but have also been identified as an early risk factor for mortality [4,5]. The pathophysiology behind this increased risk is thought to involve vasculopathy and focal hypoperfusion, which are known complications of the disease [6]. Despite these established associations, there remains limited understanding of the impact of SCD as a comorbidity on seizure-related hospitalizations, particularly in terms of demographic disparities, clinical characteristics, and outcomes. Addressing these gaps is essential to improving the care and prognosis of individuals with SCD who experience seizures.

## Materials and methods

Study design and database description

This study utilized the 2021 National Inpatient Sample (NIS), a comprehensive, all-payer database developed by the Healthcare Cost and Utilization Project (HCUP) under the Agency for Healthcare Research and Quality (AHRQ). The NIS includes data from 48 participating states, representing 97% of the US population and 96% of discharges from the US community hospitals, excluding rehabilitation and long-term acute care facilities. It approximates a 20% stratified sample of discharges, ensuring representativeness based on hospital characteristics such as ownership, bed size, teaching status, and location. The self-weighting design reduces the margin of error, providing stable and precise national estimates while protecting patient confidentiality by excluding state and hospital identifiers. Its large sample size offers substantial statistical power to detect differences across patient populations and outcomes, making it a valuable resource for healthcare research. This study used publicly available, de-identified data from the NIS, which does not require Institutional Review Board (IRB) approval as per the guidelines of the HCUP. Because no identifiable private health information was used, this study was exempt from IRB review.

Study population

The study included all patients admitted with a principal diagnosis of seizures, identified using the International Classification of Diseases, Tenth Revision (ICD-10) codes provided in the supplementary table. The study population was divided into two groups based on the presence or absence of SCD as a comorbidity. We analyzed patient demographics and hospital characteristics, comparing these factors between the two groups to evaluate differences in outcomes.

Statistical analysis

Data analysis was conducted using Stata Statistical Software version 18 (StataCorp LLC; College Station, TX). T-tests were used to calculate p-values for continuous variables, while Fisher’s exact test was applied to binary and categorical variables. Multivariate logistic regression analysis was performed to assess the association between the comorbidity of SCD and binary outcomes, while multivariate linear regression was used for continuous outcomes. Both models were adjusted for potential confounders to ensure robust and reliable results.

Outcomes

The primary outcome of interest was in-hospital mortality. Secondary outcomes included total hospital charges and total length of stay. 

## Results

Patient demographics and hospital characteristics

The NIS dataset included 33.3 million observations, of which 263,625 were adult patients admitted with a principal diagnosis of seizures. Among these, 434 (0.17%) had a comorbid diagnosis of SCD. Table 1 provides a detailed comparison of patient demographics and hospital characteristics for patients with and without SCD diagnosis.

**Table 1:** Patient demographics and hospital characteristics

	No sickle cell disease	Sickle cell disease	p-value
Female (%)	120,000 (47.33)	260 (59.09)	0.04
Mean age (95% conf. interval)	44.1(42.89-45.3)	39.02(34.97-43.07)	0.01
Race/ethnicity (%)			<0.01
White	140,000 (56.65)	30 (7.06)	
Black	58,000 (22.76)	335 (78.82)	
Hispanic	35,000 (13.74)	15 (3.53)	
Asian or Pacific Islander	5320 (2.09)	0 (0.00)	
Native American	1950 (0.77)	0 (0.00)	
Other	10,000 (3.99)	45 (10.59)	
Median annual income in patient’s zip code, US$, no. (%)			0.26
$1-$51,999	84,000 (32.58)	160 (37.65)	
$52,000-$65,999	63,000 (24.45)	130 (30.59)	
$66,000-$87,999	59,000 (22.86)	70 (16.47)	
$88,000 or more​	52,000 (20.1)	65 (15.29)	
Insurance type, no. (%)			0.14
Medicare	92,000 (36.42)	125(28.74)	
Medicaid	81,000 (32.04)	185 (42.53)	
Private insurance, including Health Maintenance Organizations (HMO)	67,000 (26.56)	95 (21.84)	
Self-pay	13,000 (4.98)	30 (6.9)	
Hospital region, no. (%)			<0.01
Northeast	55,000 (20.9)	115 (26.14)	
Midwest	58,000 (21.94)	70 (15.91)	
South	1000,000 (38.84)	235 (53.41)	
West	48,000 (18.33)	20 (4.55)	
Urban location (%)	250,000 (96.03)	440 (100)	0.11
Teaching hospital	220,000 (84.44)	375 (85.23)	0.84

Significant differences were observed between the two groups. The sickle cell group had a higher proportion of women (59.09% vs. 47.33%, p < 0.05) and was, on average, younger (mean age 39.02 years vs. 44.1 years, p < 0.05). There is a markedly higher proportion of African American patients in the sickle cell group (78.82% vs. 22.76%, p < 0.05), consistent with the established epidemiology of the disease.

No significant differences were found in the median annual income of the patients' ZIP codes or insurance types between the groups. However, a significantly larger proportion of patients with SCD were hospitalized in the South (53.41% vs. 38.84%, p < 0.05). The distribution across urban settings and teaching hospitals was comparable between the groups, with no statistically significant differences observed. These findings underscore the demographic and regional disparities associated with SCD and highlight its prevalence among younger, African American populations, predominantly in the South.

Distribution of clinical characteristics

Patients with SCD exhibited notable differences in clinical characteristics compared to those without the condition. A significantly higher proportion of patients in the sickle cell group experienced ischemic stroke (4.55% vs. 1.16%, p < 0.05). Additionally, the Charlson Comorbidity Index (CCI) scores were markedly higher among the sickle cell group, with a greater proportion having scores of ≥3 (31.82% vs. 18.79%, p < 0.05), indicating a higher burden of comorbidities. No statistically significant differences were observed between the groups in the occurrence of status epilepticus (22.73% vs. 17.13%, p = 0.17), non-traumatic intracerebral hemorrhage, traumatic brain injury (TBI), or meningitis. Table 2 provides a comprehensive comparison of these clinical characteristics.

**Table 2:** Relevant clinical characteristics TBI: traumatic brain injury

	No sickle cell disease	Sickle cell disease	p-value
Patients with a principal diagnosis of status epilepticus (% of total seizure admission)	45,000 (17.13)	100 (22.73)	0.17
Comorbidities			
Charlson Comorbidity Index score, no. (%)			0.0075
0	130,000 (50.28)	155 (35.23)	
1	49,000(18.55)	90 (20.45)	
2	33,000 (12.38)	55 (12.5)	
≥3	49,000 (18.79)	140 (31.82)	
Ischemic stroke (%)	3040 (1.16)	20 (4.55)	0.0028
Nontraumatic intracerebral hemorrhage (%)	2720 (1.03)	0 (0)	0.38
TBI (%)	4435 (1.69)	5 (1.14)	0.68
Meningitis (%)	215 (0.00082)	0 (0)	0.79

Primary outcomes

Among the 263,625 patients admitted with a principal diagnosis of seizures, 2,915 (1.11%) died during hospitalization. The unadjusted odds ratio (OR) for mortality in patients with SCD was not significantly different from those without SCD. After adjusting for potential confounders, including age, gender, race, median annual income of the patient’s ZIP code, hospital region, urban location, teaching hospital status, CCI score, presence of status epilepticus, ischemic stroke, nontraumatic intracerebral hemorrhage (ICH), traumatic brain injury (TBI), and meningitis, the odds ratio for mortality remained nonsignificant between the groups.

These findings are detailed in Table 3. Notably, similar results were observed when the analysis was restricted to factors with a p-value <0.2 in univariate analysis, supporting the robustness of the results. Overall, the presence of SCD as a comorbidity does not appear to significantly alter the risk of in-hospital mortality in patients admitted for seizures.

**Table 3:** Primary outcomes

Outcomes	Exposure	Odds ratio (OR)	95% confidence interval (CI)	p-value
Mortality (unadjusted)	Sickle cell disease	1.03	0.14-7.45	0.978
Mortality (adjusted)	Sickle cell disease	1.60	0.22-11.44	0.637

Secondary outcomes

The mean length of stay for patients admitted with a principal diagnosis of seizures was 4.2 days. Adjusted analyses showed no statistically significant difference in the mean length of stay between patients with and without SCD. Similarly, the mean total hospital charges for these patients were $57,500, with no significant differences observed in the adjusted mean total hospital charges between the two groups. These findings indicate that the presence of SCD as a comorbidity does not appear to substantially influence the duration of hospitalization or associated costs in patients admitted with seizures. Detailed results are presented in Table 4.

**Table 4:** Secondary outcomes

Adjusted secondary outcomes	Value	95% confidence interval (CI)	p-value
Mean length of stay (sickle cell disease)	0.14	-1.08-1.35 days	0.825
Total hospital charges (sickle cell disease)	$1553.54	-$12,348.98-$15,456	0.827

## Discussion

This study provides critical insights into the impact of SCD on seizure-related hospitalizations. The findings highlight the unique demographic and clinical characteristics of SCD patients, distinguishing their profiles from non-SCD populations. The results reveal several key observations regarding the role of SCD as a comorbidity in seizure-related hospitalizations. Patients with SCD were younger and predominantly female, with a significantly higher proportion identifying as African American, consistent with the known epidemiology of the disease. These patients also had a higher burden of comorbidities, as reflected in the elevated CCI scores, and were more likely to experience ischemic stroke than patients without SCD. However, despite these differences, SCD was not associated with significant changes in primary outcomes, including in-hospital mortality, length of stay, or total hospital charges, after adjusting for potential confounders.

SCD is known to be associated with a wide range of acute and chronic comorbidities affecting nearly every organ system. These comorbidities tend to increase with age, particularly the chronic ones [7]. This systemic burden was reflected in our study by the higher CCI scores observed among SCD patients compared to their non-SCD counterparts. These findings underscore the importance of comprehensive and aggressive management of comorbidities in this population to improve clinical outcomes.

The risk of seizures in SCD is multifactorial, involving stroke severity, cortical involvement, and systemic inflammation. The link between cerebral ischemia and seizures is well established in SCD, with both silent and overt cerebral infarcts serving as major contributors to seizure pathogenesis. The elevated ischemic stroke rates seen in this study are consistent with previous findings, where ischemia-induced neuronal damage and excitotoxicity have been identified as key mechanisms underlying seizure development [2,8].

Focal hypoperfusion and sickle cell vasculopathy may further increase seizure susceptibility, while cortical involvement and gliotic scarring contribute to heightened neuronal excitability, consistent with patterns seen in post-stroke epilepsy [9]. Additionally, systemic inflammation plays a central role in epileptogenesis. The inflammatory cascade, including the release of interleukin-1β and other cytokines, can disrupt the blood-brain barrier and promote neuronal hyperexcitability [10]. This is supported by broader literature on vascular disorders, in which neuroinflammation and impaired neurovascular coupling are linked to increased seizure risk [11].

Interestingly, although earlier studies identified seizures as a risk factor for increased mortality in patients with SCD, our study did not find a significant difference in in-hospital mortality among patients with seizures. This aligns with more recent mortality data, such as the 2024 SCDIC registry study, which identified cardiac causes as the most common cause of death [12]. These findings may reflect advancements in the acute management of seizures and SCD-related complications, as well as the widespread use of hydroxyurea, particularly in institutions offering specialized care. Nonetheless, the systemic burden of disease, reflected in high CCI scores, reinforces the need for comprehensive care strategies. Additionally, regional differences, such as higher hospitalization rates in the Southern United States, mirror known epidemiological trends and may point to disparities in healthcare access and resource distribution.

This study has several limitations. First, it is based on the NIS, which relies on administrative coding for discharge-level variables, assuming accurate documentation. However, the potential for misclassification of diagnoses cannot be entirely ruled out. Additionally, because the NIS captures hospital discharges rather than unique patients, it is possible that some individuals may have been included more than once if they had multiple admissions during the study period. This may slightly inflate event counts but is unlikely to systematically bias the comparative analyses. The NIS encompasses approximately 20% of all hospital discharges and uses stratified sampling and weighting methods to generate national estimates. However, as a sample rather than a complete census, certain patient or hospital-level characteristics may be underrepresented, which could affect the generalizability of our findings to the broader US inpatient population. As a retrospective study, the analysis is inherently limited in its ability to establish causal relationships. Additionally, the dataset lacks clinical granularity, including information on seizure severity, electroencephalogram (EEG) findings, and specific treatment details. This limitation restricts our ability to evaluate important clinical aspects of seizures, such as their type, severity, and responsiveness to treatment, which may influence outcomes. Selection bias may be present due to variations in hospital reporting and coding practices. Furthermore, unmeasured confounders, such as socioeconomic status, access to outpatient care, and medication adherence, could influence patient outcomes. While the relatively small number of patients with SCD may have limited our ability to detect subtle differences in outcomes, the size of the overall cohort and the resulting adjusted analyses likely had sufficient power to detect large, clinically significant differences. The absence of such findings, combined with the direction and magnitude of our effect estimates, suggests that any potential differences are likely to be modest. As such, these findings should be interpreted in that context, and future studies with larger SCD-specific cohorts may help confirm these results. Despite these limitations, the large sample size and national representation provide valuable insights into the impact of SCD on seizure-related hospitalizations.

## Conclusions

The short-term outcomes of SCD patients hospitalized for seizures are comparable to those without SCD. Further research is needed to evaluate the long-term outcomes of seizure-related hospitalizations in SCD patients, including neurological recovery and quality of life.

## Appendices

**Table 5:** ICD 10 codes ICD: the International Classification of Diseases, Tenth Revision; ICH: intracerebral hemorrhage; TBI: traumatic brain injury

Diagnosis	ICD 10 codes
Seizures	G40x, G41x, and R56x
Status epilepticus	G41.0, G41.1, G41.2, G41.8, G41.9, G40.A01, G40.A11, G40.B01, G40.B11, G40.401, G40.301, G40.311, G40.411, G40.201, G40.211, G40.001, G40.101, G40.011, G40.111, G40.501, G40.801, G40.803, G40.811, G40.813, G40.823, G40.821 G40.833, G40.901, G40.911
Sickle cell disease	D5700, D5701, D5702, D5703, D5704, D5709, D571, D5720, D57211, D57212, D57213, D57214, D57218, D57219, D5740, D57411, D57412, D57413, D57414, D57418, D57419, D5742, D57431, D57432, D57433, D57434, D57438, D57439, D5744, D57451, D57452, D57453, D57454, D57458, D57459, D5780, D57811, D57812, D57813, D57814, D57818, D57819
Meningitis	G000, G001, G002, G003, G008, A390, A170, A321, A521, A543, A232, A203, A278, A373, A870, A871, A872, A878, A879, B003, B010, B021, B330, A821, B375, B451, B483, B583, B675, B698, B718, B721, B738, G01, G02
Ischemic stroke	I63x
Nontraumatic ICH	I60x, I61x, I62x
TBI	S06x

The Charlson Comorbidity Index (CCI) is a validated scoring system used to quantify a patient’s comorbidity burden and predict the risk of mortality based on the presence and severity of 17 medical conditions. Each condition is assigned a weighted score (ranging from 1 to 6) depending on its association with mortality risk. The total CCI score is the sum of these weights, with higher scores indicating a greater burden of comorbid disease and an increased likelihood of adverse outcomes, including death.

CCI-17 Conditions

**Table 6:** Charlson Comorbidity Index-conditions and weights

Comorbidity	Weight
Myocardial infarction	1
Congestive heart failure	1
Peripheral vascular disease	1
Cerebrovascular disease	1
Dementia	1
Chronic pulmonary disease	1
Connective tissue disease	1
Peptic ulcer disease	1
Mild liver disease	1
Diabetes without chronic complications	1
Diabetes with chronic complications	2
Hemiplegia or paraplegia	2
Renal disease	2
Any malignancy (excluding nonmelanoma skin)	2
Moderate or severe liver disease	3
Metastatic solid tumor	6
AIDS/HIV	6
